# Best practices, challenges and innovations in pediatrics in 2019

**DOI:** 10.1186/s13052-020-00941-1

**Published:** 2020-11-30

**Authors:** Carlo Caffarelli, Francesca Santamaria, Angelica Santoro, Michela Procaccianti, Fabio Castellano, Francesca Fiori Nastro, Alberto Villani, Sergio Bernasconi, Giovanni Corsello

**Affiliations:** 1 Clinica Pediatrica, Department of Medicine and Surgery, Azienda Ospedaliera-Universitaria, University of Parma, Via Gramsci 14, Parma, Italy; 2 Department of Translational Medical Sciences, Federico II University, Naples, Italy; 3 UOC di Pediatria Generale e Malattie Infettive, Ospedale Pediatrico Bambino Gesù, Rome, Italy; 4 Microbiome Research Hub, University of Parma, Parma, Italy; 5 Department of Sciences for Health Promotion and Mother and Child Care “G. D’Alessandro”, University of Palermo, Palermo, Italy

**Keywords:** Allergy, Endocrinology, Critical care, Endocrinology, Gastroenterology, Infectious diseases, Neonatology, Neurology, Nutrition, Children, Respiratory diseases

## Abstract

This paper runs through key progresses in epidemiology, pathomechanisms and therapy of various diseases in children that were issued in the Italian Journal of Pediatrics at the end of last year. Novel research and documents that explore areas such as allergy, critical care, endocrinology, gastroenterology, infectious diseases, neonatology, neurology, nutrition, and respiratory tract illnesses in children have been reported. These observations will help to control childhood illnesses.

## Introduction

This review reports key advances in allergy, critical care, endocrinology, gastroenterology, infectious diseases, neonatology, neurology, nutrition, and respiratory tract illnesses in children. Articles have been identified among the most accessed manuscripts issued in the Italian Journal of Pediatrics in the second semester of 2019.

## Allergy. 1- chronic urticaria; 2- cow’s milk allergy; 3- type 1 asthma

Chronic urticaria continues to have a high prevalence in children [1], but knowledge about management is low in real-life [2]. The updated guideline for clinical management of chronic urticaria in children [3] is intendent to facilitate physicians’ work. Progresses that has been made in understanding the pathophysiology have been focused. The guideline underlines that chronic urticaria is diagnosed by history, examination, and specific investigations and it also provides a diagnostic algorithm. Regarding treatment, second generation anti-H1 antihistamines are the treatment of choice and the addition of corticosteroids may improve control of exacerbation [4]. Novel approaches such as omalizumab, have been found to be highly effective and safe [5].

Further developments are pertinent to allergy. Cow’s milk protein allergy is the most common food allergy in young children, especially those who suffer from atopic dermatitis [6] while allergy to fruits and vegetables is the more frequent in school children [7]. History, skin tests, oral food challenge and specific IgE antibody measurement are used for the diagnosis [8, 9]. The standard treatment is based on avoidance of offending food and education about treatment of reactions. Cow’s milk has essential nutritional properties, especially for infants. Thus, adequate substitutes are warranted. Extensive hydrolysate of cow’s milk proteins or rice, and amino acid formulas are hypoallergenic and ensure growth and development. Soy formulas can be considered after 6 months of age. However, less expensive, and more palatable formulas would welcome. Some mammalian milks with better taste have been proposed as infant formulas, but they are not recommended because of nutritional concerns. Among them, donkey’s milk has weak clinical cross-reactivity with cow’s milk proteins, but it is costly and hypocaloric with a high mineral content compared to human milk. Sarti et al. [10] looked at a novel approach. They performed oral challenges with donkey’s milk in 70 children with IgE-mediated cow’s milk allergy aged 6 months − 18 years of age and 11 with food protein-induced enterocolitis syndrome (FPIES) to cow’s milk age range 3 months – 8 months. Only one child with IgE-mediated cow’s milk allergy reacted. Sixteen out of the 70 patients with IgE- cow’s milk allergy and 6 out of the 11 patients with FPIES to cow’s milk followed a diet with donkey’s milk and adequate supplements including lipids and Vitamin D. All patients had normal growth, a valuable addition to current knowledge.

Precision medicine targets the mechanisms (endotypes) of allergic diseases. In asthma, the precision approach is based on monoclonal antibodies [11] and allergen specific immunotherapy [12]. Giovannini et al. [11] compared omalizumab and mepolizumab. They are monoclonal antibodies used in type 2-high asthma [13]. Omalizumab is a humanized monoclonal IgG antibody binding the circulating IgE for children with moderate - severe persistent allergic asthma to perennial aeroallergens that is not controlled with inhaled corticosteroids and long-acting inhaled β2-agonist. It is useful also when seasonal pollens [14] induce exacerbations [11]. Mepolizumab is a humanized monoclonal IgG antibody binding IL-5 for add-on treatment in asthmatics with elevated blood eosinophils count. Efficacy and safety of omalizumab and mepolizumab did not differ significantly. Mepolizumab is effective in asthmatics who did not previously responded to omalizumab. More comparative data on their efficacy, long-term safety, cost/benefit ratio in children who are eligible for both drugs, are needed.

## Critical care. 1- dental trauma; 2- pain

The incidence/year of dental trauma in children is 4.5%. It might occur in many different settings: home, school, sport settings or during free-time activities. So, it is necessary for pediatricians, teachers, sport staff to have guidelines to properly manage facial trauma in children. The updated Italian guideline [15] provides indication about prevention of dental trauma, diagnosis approach, management, and medico-legal implications. The correct approach in orofacial and dental trauma includes a physical examination, especially intra and extra-oral examination. When a bone fracture is suspected, the radiological investigation of the facial district is necessary to confirm or exclude the fracture. Therapeutic approach is multidisciplinary: maxillofacial surgeon and dentist depending on the facial district involved. Nevertheless, an urgent first aid must be guaranteed to avoid adverse outcome. Prognostic factors need to be carefully reported at both initial consultation and during the follow up visits [16]. Attention must be paid also to medicolegal aspect of the injury; a certificate of dental trauma should be appropriately filled. Moreover, when dental injury is suspected to be the result of an abuse or violence against children, it must be reported to authorities. In a recent study, Bossù et al. [17], evaluated the application of National guidelines for prevention and clinical management of traumatic dental injuries in developmental age and found that they are not uniformly applied. If prevention strategies are correctly applied, many dental injuries might be avoided, moreover, a proper management of trauma can improve the outcome.

Virtual reality (VR) is a computer-generated simulating experience designed to make it as real as possible. Fields of application include entertainment and educational purposes such as video games and surgery or military training. In terms of clinical application, VR has also been investigated as a therapeutic tool, in particular for treating phobias, social disorder, and pain. Iannicelli et al. [18] analysed the efficacy of VR as an instrument for pain reduction in children and adolescents. Although there are a few publications in this field, VR seems to be effective for non-pharmacological pain reduction. In last years, several studies investigated the use of VR for reducing acute pain during various medical procedures, while there are no studies regarding chronic pain and only one case report [19] described the use of VR in palliative care. Along this line, Eijlers et al. [20] has published the first systematic review and meta-analysis specifically focused on VR in pediatric patients showing that VR diminished patient-reported pain and anxiety during a range of medical procedures. Despite the use of VR in reducing pain in pediatric patients seems encouraging, more studies, both on acute and chronic pain, are needed to better define his possible role in standard care. Moreover, commercial products lack variety and meaningful design strategies [21]. The collaboration with VR designers would be of interest to create applications tailored for children.

## Endocrinology. 1- growth hormone; 2- turner syndrome; 3- preterm growth

Growth impairment is a common feature in mucopolysaccharidoses patients, regardless of type or severity. There is a however a continuous spectrum of phenotypic expression from the very severe to the mild, with variable ages of onset and rates of progression. Moreover, the adult height attained depends on phenotype severity [22]. Growth impairment seems due to the deposition of glycosaminoglycans in bone and cartilage which leads to progressive damage in cartilage that in turn reduces bone growth by destruction of the growth plate and incomplete ossification. However, the mechanism behind short stature is not completely known. Structurally, skeletal abnormalities limit longitudinal growth and final height, but alone cannot explain short stature [23]. Standard therapy based on enzyme replacement therapy and hematopoietic stem cell transplantation can improve the progression of the disease, particularly when started at young age, but its effect on the growth seems to be limited. It would therefore be useful to find ways to improve adult stature. Human growth hormone (hGH) therapy in unselected patients has not been proven to be effective [23]. However, in a case series of mucopolysaccharidoses patients of different type with growth hormone deficiency (GHD) Cattoni et al. [24] concluded that hGH therapy appears to have effectively reverted the growth deceleration, at least during the first 12–24 months of treatment without any evident side effects. Various points derive from this interesting study which deserve to be further investigated. In particular: a) what is the prevalence of GHD in the different forms of mucopolysaccharidoses? In the experience of these Authors, it appears to be high involving 6 out of 64 patients; b) what is the mechanism of involvement of the GH-IGF-1 axis? It is interesting to note that the scarce data available are contradictory, indicating, for example, in Hurler’s syndrome, a high GH value in more than half of the patients [25]; c) how the hypothalamus-pituitary axis of different hormones may be involved? Gonadotropin axis could be altered as demonstrated by lack of puberty in some patients [25]; d) what is the influence on the final height of the response to GH therapy? e) what is the long-term safety? In conclusion, the experience of Cattoni et al. [24] stimulates us to investigate the changes that mucopolysaccharidoses can produce at the endocrine level and to better identify patients who could benefit from GH therapy. On the other hand it is important to remember that other therapeutic approaches are in the experimental phase such as the recent report of a positive effect in the experimental animal of the addition of C-type natriuretic peptide to enzyme replacement therapy.

The report by Chen et al. [26] of a second trimester intrauterine foetal demise of a foetus with septated nuchal lymphatic hygroma and hydrops fetalis, in which a 45XO karyotype was highlighted, allows to underline some aspects of interest for clinical practice:

- The cystic hygroma of the neck is a malformation due to an accumulation of fluid in the lymphatic vessels and jugular sacks due to an altered connection between the lymphatic and venous systems. It is mono or multiloculated and is diagnosed with sonography already in the first trimester of pregnancy
- Prenatal cytogenetic analyzes showed in 30–60% of cases the association with aneuploidy and in particular with 18 and 21 trisomies and with Turner syndrome. According to some experiences, aneuploidy is more frequent with septated than non-septated cases [27].
- In cases with normal karyotype there are still major malformations, especially cardiac but also urogenital, skeletal, nervous system and body wall anomalies in up to one-third of foetuses. Furthermore, by genomic microarray, it was found that in 5% of foetuses there is a pathogenic genomic imbalance and the most frequently found is 22q11.2 named DiGeorge syndrome [28]. Finally, some AA recommend prenatal testing of PTPN11, KRAS and RAF1 in pregnancies with an increased NT and at least one of the following additional features: polyhydramnios, hydrops fetalis, renal anomalies, distended JLS, hydrothorax, cardiac anomalies, cystic hygroma and ascites. If possible, mutation analysis of BRAF and MAP 2 K1 should also be considered
- If hydrops develops in addition to the hygroma, as in the case described, prognosis is detrimental,
- The presence of partial, cutaneous syndactyly involving digits 2–5 of the fingers and toes is very rare and does not yet find a precise explanation. It is however interesting to note that according to Mazzaschi et al. [29] syndactyly has been described in patients with a cell line containing a small marker of X Chromosome origin .

Factors affecting the catch-up growth in preterm infants are an important clinical issue [30]. Liu et al. [31] in their interesting article address the important aspect of recovery growth of preterm infants after discharge. The authors based their analysis on the health belief model, a social psychological health behavior change model developed to explain and predict health-related behaviors. The health belief model suggests that people’s beliefs about health problems, perceived benefits of action and barriers to action, and self-efficacy explain engagement (or lack of engagement) in health-promoting behavior [32]. In this study four dimensions of post discharge feeding knowledge and six dimensions of feeding-related health beliefs scores of preterm infants’ caregivers were considered and scored. Then, a logistic regression analysis on several factors influencing the catch-up growth of preterms infants after discharge has been conducted. Studies involving preterm infants showed that decreased gestational age and birth weight, increase the incidence of extrauterine growth restriction [33]. In comparison to two cited previous studies on postnatal growth pattern of preterm newborns, in this study the proportion of extremely low-birth-weight infants was lower (1.3%). A catch-up growth, at 12 months corrected age, has been not achieved in 17.4%, and was related to gestational age, regular health care, mothers’ daily contact with the baby, caregivers’ educational background, monthly average family income, and addition of breast milk supplements. Being small for gestational age and very low birth weight have been recently confirmed as risk factors for poor growth in another study from China [34]. Key suggestions are given to increase post discharge catch-up growth of preterms infants. Among them, close post discharge health monitoring and changes in social policies aimed at an increased length of maternity leave, are of greater importance. Interestingly, this study showed that the health beliefs of caregivers had no significant effect on the catch-up growth of preterm infants.

## Infectious diseases. 1- hand washing; 2- enterovirus; 3- osteomyelitis; 4- vaccines

Infections are one of the major causes of infant mortality in developing countries. Human hands represent an important vehicle of pathogen transmission by touching nose, mouth, or eyes. Dagne et al. [35] performed a questionnaire community based cross-sectional study in Debark town (Ethiopia). A good hand washing practice was found in 52% of mothers of under five children and the most significant associated factors were knowledge, attitude, and water availability. In another study conducted in Northwest Ethiopia [36]. Mothers had good handwashing knowledge in 66.05% of instances. Children who had mothers with good handwashing knowledge were 51% less likely to have diarrhea than those with mothers of poor knowledge. Literacy, level of education, economic status, and social norms played a crucial role in preventing children infections. The results of these studies are confirmed by Taddese et al. [37] who assessed handwashing in under-five children admitted at an Ethiopian hospital. Handwashing was correctly performed by 39.1% of mothers. Poor hygienic practices can influence diarrheal admissions among under-five children.

Enteroviruses are RNA viruses belonging to the family Picornaviridae, that are transmitted from person to person via fecal-oral or respiratory routes. Enteroviral infections are spread worldwide and show a seasonal pattern with a peak during summer. Clinical manifestation may vary according to the age of presentation [38] from asymptomatic or mild and self-limited disease to more severe infections especially in neonates and young infants. Severe enteroviral diseases in neonates include hepatic necrosis with coagulopathy, meningoencephalitis, and myocarditis [39]. Moreover, enterovirus is one of the primary causes of central nervous system infections in childhood [40].

Berardi et al. [38] analyzed clinical manifestation of enteroviral infection comparing infants younger and older than 90 days. They found that fever was common in both groups, cutaneous rash and seizure were predominant among younger children, whereas neck rigidity, vomiting and diarrhea were more frequent among older ones. Moreover, cerebrospinal fluid pleocytosis and increased white cell count were more characteristic of older infants.

Knowledge of the clinical syndromes allows physicians to early identify potential severe cases and to perform diagnostic tests to readily start appropriate treatment [39]. Since enteroviral central nervous system infection may cause possible neurologic sequelae, neurodevelopmental follow-up should be planned in these patients [38].

Brodie’s abscess is a subacute hematogenous osteomyelitis characterized by a low-grade pyogenic abscess with milder clinical features than the acute form. The treatment of Brodie’s abscess may vary. Successful treatment is frequently achieved with prolonged parenteral administration of antibiotics associated with immobilization, though thus some patients require curettage [41]. Local gentamicin-PMMA beads implantation can also be used. Cossio et al. [42] described a 2-years-old male with Brodie’s abscess of the distal diaphysis of tibia. First, he unsuccessfully treated with oxacillin for 20 days. He then received rifampicin and levofloxacin that was immediately halted because of allergic reactions. Subsequently, trimethoprim sulfamethoxazole was introduced and then interrupted because of leukopenia, followed by oral rifampicin and doxycycline. After 2 months of treatment, magnetic resonance imaging showed no improvement of the bone lesion. The patient underwent to surgical treatment with bioactive glass BAG-S53P4, a bone substitute with antibacterial properties and a good safety profile. Seventeen months later the patient was free of infection and presented a complete recovery. The BAG-S53P4 did not affect the growth. However, it is important to extend the clinical and the radiographic follow up through the weight-bearing radiographs in children with sub-acute osteomyelitis of the lower limb since Brodie’s abscess can result in growth deformities [43]. These problems may also develop many years after the bone injury.

Healthcare workers (HCWs) have an important role in informing, advising, and promoting vaccinations. They are also particularly exposed to vaccine-preventable diseases and their vaccination is important to reduce disease transmission to other members of staff and their patients, particularly the vulnerable groups. Tomboloni et al. [44] performed an observational study with the aim to detect the knowledge, skills, attitude, and barriers of HCWs regarding vaccination in a tertiary children’s hospital in Italy. At variance from other recent studies [45] who identify gaps in knowledge as the leading cause of the decrease of vaccination coverage among HCWs, Tomboloni et al. suggest that this effect is due to a low perception of risk associated with the disease rather than lack of knowledge. However, their statement is only based on data regarding flu vaccine. Along the same lines Di Martino et al. also considered the main reason for missing flu vaccination was the presumed uselessness of the vaccine [46]. In another research [47] it was observed that the anti-influenza vaccination coverage of HCWs in Italy is far below the minimum targets set. The authors consider the informative role of the occupational physicians as fundamental to increase the awareness of the potential of the flu vaccine. Di Martino et al. [46] also suggest taking advantage of COVID-19 epidemic to sensitize the population and the HCWs about flu vaccination. In particular, flu vaccination could facilitate the differential diagnosis in cases of new COVID-19 outbreak during the next winter season. So, the Italian Government strongly recommended the flu vaccine in 2020 for the pediatric population between 6 months and 6 years of age in addition to the other risk category.

The role of HCWs in promoting vaccination is fundamental in preterm infants, who due to their immature immune system, are more vulnerable to vaccine-preventable disease. It has been shown that preterm infants, regardless of their degree of prematurity, have low vaccination rates and delays [48]. Possible reasons [48] include parental and provider perception of medical vulnerability, lack of information on vaccine recommendation and concerns about vaccine safety [49]. Hexavalent vaccines protect against diphtheria, tetanus, pertussis, poliovirus, hepatitis B virus, *Haemophilus influenzae* and are routinely administered in most European countries. Hexavalent vaccines in Italy should be administered in preterm infants with the same schedule of term babies [50–52]. An updated joint document by Italian Society of Pediatric Allergy and Immunology jointly with the Italian Society of Neonatology on hexavalent vaccines in preterm infants has been published [50]. Regarding the safety of hexavalent vaccines, an increased incidence of apnoea, bradycardia, desaturation occurs in preterm infants in 48 h following injection. Apnoea episodes develop in preterm infants with most severe clinical conditions who required continuous positive airway pressure, and who have had similar episodes, especially within 24 h prior to the injection. Therefore, preterm babies who are still hospitalized should receive the first dose of vaccine during the hospitalization, particularly if they present risk factors for adverse events [50]. Similarly, in the most severe preterm infants (< 28 weeks) or those with a recent history of respiratory distress, vaccine should be administered at hospital and patients should be monitored for 2 or 3 days. Risk factors for recurrence of post-vaccination apnoea are birth weight < 2 kg, gestational age ≤ 31 weeks, age < 67 days, previous similar episodes, and hospitalization for condition associated with prematurity. Infants at risk of recurrence should get the second dose under medical supervision in a setting with facilities for treating adverse reactions and should be monitored for 48–72 h.

## Gastroenterology. 1- cholestatic jaundice

The clinical diagnosis of neonatal cholestasis is considered an extremely challenging process. In their commentary, Mandato et al. [53] have highlighted not only the importance of a prompt distinction between extrahepatic and intrahepatic cholestasis, but also underlined the role of the correct identification of the latter amongst the hotchpotch of the recently discovered metabolic/genetic causes.

The diagnosis is based on anamnestic and clinical elements, as well as on laboratory and instrumental data. As far as the clinical features, in addition to jaundice, patients may present with symptoms or signs that are considered as red flags including hyperchromic urines, hypocholic or acholic stools, and incipient itching. For the laboratory evaluation, the American Academy of Pediatrics and the North American and European Associations for Pediatric Gastroenterology, Hepatology and Nutrition recommend that infants with jaundice lasting more than 2–3 weeks are tested for both serum conjugated and total bilirubin, and are evaluated by an expert as soon as possible [54, 55].

Neonatal cholestasis may stem from several conditions. The archetypal types of exclusive/prevalent extrahepatic cholestasis are biliary atresia and choledochal cyst [56]. Biliary atresia is considered a surgical emergency in newborns. The rate of success in establishing bile drainage is in fact a function of the early age when the hepato-portoenterostomy intervention is made. Intrahepatic cholestasis is due to a broad and more and more puzzling variety of infectious, endocrine, genetic, metabolic, and toxic disorders, whereas the gamma-glutamyl transpeptidase serum levels may help for the differential diagnosis. Recently established laboratory diagnostic techniques have allowed to discover new causes of neonatal cholestasis. In this regard, the group of Pinon and co-workers reported on another syndromic condition characterized by cholestasis, due to a hepatic picture of paucity of intralobular bile ducts combined with renal involvement and paved the way to the inclusion of the hepatocyte nuclear factor-1-beta deficiency as a new condition to be considered in the diagnostic process of the syndromic forms with paucity of intralobular bile ducts [57].

## Neonatology. 1- neonatal emergency transport; 2- skin-to-skin contact; 3- non-invasive ventilation; 4- low birth weight; 5-sepsis

The benefits and effects of skin-to-skin contact and breast-feeding immediately after birth, are widely known. They include an enhanced frequency and length of breast feeding, the maintenance of glycemia and temperature, the cardio-respiratory stability in preterm newborns, the improved maternal attachment behaviour and decreased crying [58]. However, early skin-to-skin contact is rarely associated with sudden unexpected postnatal collapse that can have serious outcomes [59]. The importance of early skin-to-skin contact after birth, as well as the inherent risk factors that may cause sudden unexpected postnatal collapse led to the necessity of evaluating how reduce the risk of its occurrence. Barbaglia et al. conducted a questionnaire study on skin-to-skin contact and delivery room practices in a longitudinal survey which compared hospital data collected in 2012 and in 2016 in 2 regions of Northern Italy [60]. The survey showed the efforts in promoting early skin-to-skin contact after birth in the wards. However, it highlighted several aspects that favour the occurrence of sudden unexpected postnatal collapse. Regulation of the timing and length of skin-to-skin contact and how it was performed were lacking. Vital signs during early skin-to-skin contact were routinely recorded in only about 30% of wards in 2012, in 50% in 2016. Written procedures were available in a minority of wards (12/28 in 2012 and 10/26 in 2016). Mother-baby dyads were continuously observed in nearly half of the wards. Authors believe that surveys at regular intervals may permit identifying changes to be made to have safer skin-to-skin care birth centres.

Until now, the European Community rules have specified the requirements for the design, testing, performance, and equipping of the road ambulance used for the transport and care of injured or ill adults, completely ignoring the neonatal transport. Bellini et al. describe in detail a novel project aimed to realize a dedicated ground ambulance for the neonatal emergency transport service [61] avoiding fitting an ambulance with superfluous devices for adult use. They proposed that the cabinets should contain essential equipment for the neonatal transport, such as gloves and catheters box, the intubation set, or central catheters, that are usually stored into the transport bag, thus being immediately available and easily transportable. They demonstrated that it is not possible to simply adapt the currently dedicated ambulance for mobile intensive care and resuscitation services (actual type C European Community) in a modern ambulance for emergency transport service. It is of paramount importance that the European Community introduce an additional ambulance type, the type D, exclusively reserved to transport neonates. Main differences between the European Type C ambulance and the new proposed Type D neonatal ground ambulance are the presence on board of several devices such as the air compressed cylinder, iNO cylinders and delivery system, the phototherapy lamp, shock adsorbing stretcher support, cooling device, a placenta refrigeration box and the transcutaneous gas analyzer [61]. The current ambulance configuration is far to the proposed ambulance design. The hope is to re-think and re-design the available ambulance models to eventually integrate the EU regulations adding the D ambulance model which is reserved to the neonatal transport.

The deleterious consequences of the management of neonatal respiratory distress syndrome (RDS) by invasive ventilation have led to more in-depth investigation of non-invasive ventilation (NIV) modalities. In their review, Permall et al. [62] described and compared several different modalities of NIV currently available, underlining strengths and limitations of each of them. Despite its benefits in terms of survival of preterm infants, invasive mechanical ventilation for the treatment of neonatal respiratory disease has also resulted in an increased number of cases with bronchopulmonary dysplasia (BPD) [63]. NIV has significantly and positively modified the treatment outcomes and significantly improved the mortality rates of preterm infants with RDS [64]. Among the NIV modalities, the nasal intermittent positive pressure ventilation (NIPPV) has shown considerable benefits compared to nasal continuous positive airway pressure (NCPAP) [65]. However, in one of the largest studies ever published, Kirpalani et al. reported that NIPPV did not prove to be superior to continuous positive air pressure (CPAP) in extremely low birth weight (ELBW) infants born before 30 weeks of gestation when outcomes such as survival with BPD or death were considered [65]. A recent addition to the NIV family in the neonatal intensive care unit is the heated humidified high-flow nasal cannula (HHHFNC), which delivers heated and humidified gas through HFNC system [66]. Despite reports of HHHFNC non-inferiority compared to NCPAP, as shown by the HUNTER trial in Australia [67], other trials have been terminated due to high treatment failure rates with HHHFNC [68, 69]. Moreover, RDS management with the combination of the INSURE (INtubation SURfactant Extubation) technique and NIV ensures higher success rates [70]. One of the latest innovative method to deliver NCPAP is the neurally adjusted ventilatory assist (NAVA) that can be used invasively and non-invasively in spontaneously breathing infants [71]. It is patient-controlled and is based on diaphragm electrical activity to deliver synch. Although the long-term effects of NIV-NAVA are still unknown, it is potentially one of the NIV modes that may surpass the standard respiratory support strategies in the next future.

Low birth weight (LBW) is one of the most important risk factor for perinatal mortality, especially in developing countries [72]. In a cross-sectional study, Mekie et al. described the magnitude of LBW and maternal risk factors among women who delivered in Debre Tabor Hospital, Ethiopia [73]. The LBW prevalence was 12.0% [95%, CI: (8.5, 15.2%)]. It was similar with the prevalence found in the Amhara Region, Ethiopia [74] but lower than that of the Ethiopia Demographic and Health Survey (EDHS) [75]. Place of residence, status of pregnancy, and hemoglobulin < 11 mg/dl [58] were found to be significant associated with LBW [76] while women who lived in urban areas, who had planned pregnancy, had lower risk of delivering LBW babies. Limitations of the study include the cross-sectional design, in which recall bias may occur, the involvement of one health facility with small sample size which might affect generalizability. An integrated approach should be followed to reduce the prevalence, morbidity and mortality related to LBW.

Neonatal sepsis is one of the leading causes of neonatal morbidity and mortality especially in developing countries. Alemu et al. [77] investigated the determinants of neonatal sepsis in 82 neonates with sepsis (cases) with their index mothers and 164 neonates who had no sepsis (controls) with their index mothers. Results show that both maternal and neonatal-related factors have a significant effect on the risk of neonatal sepsis. This study reveals that while the premature rupture of membrane was found to be an obstetric-related determinant of neonatal sepsis, birth at gestational age less than 37 weeks, not crying immediately at birth, and resuscitation at birth were neonatal-related risk factors of neonatal sepsis. According to this study, preterm delivery is one of the most significant risk factors of neonatal sepsis as it is 6.9 times more likely when compared to term delivery. Moreover, neonatal sepsis among neonates who not immediately crying at birth were 2.85 times more likely as compared to those who were crying at birth [77]. This finding was in line with the previous findings in Ghana [78]. Newborn might be unable to cry due to interference of respiration [79]. The absence of breathing and/or crying might lead health care professionals to do resuscitation that is significantly associated with neonatal sepsis [80]. Indeed, since the study was done on admitted newborns, results might lack generalizability to the entire population. However, it was confirmed that infection prevention strategies need to be strengthened and/or implemented by providing special attention to the specified determinants [77].

Regarding the prevention of neonatal sepsis, probiotics have been suggested to be of benefit [81]. In a double-blinded randomized controlled study in formula-fed preterm infants, Cui et al. [82] demonstrated that *Lactobacillus reuteri* DSM 17938 did not reduce occurrence of sepsis, localized infection or necrotizing enterocolitis. However, it significantly reduced both severity and number of gastroesophageal reflux episodes, duration of full enteral feeding, improved early feeding tolerance and increased body weight, length, and head circumference. They also confirmed [83] that *Lactobacillus reuteri* enhanced daily defecation frequency and reduced hospital stays, without adverse effects. This is worthwhile since preterm infants have poor sucking and swallowing abilities, immature digestive systems, insufficient gastrointestinal motility, and low gastrointestinal mucosal barrier function [84]. Moreover, they are prone to feeding intolerance such as regurgitation, vomiting, abdominal distension, and gastric retention during feeding. A prominent condition among preterm infants is gastroesophageal reflux. When severe, symptoms such as apnoea, bradycardia, vomiting, poor weight gain and irritability have been attributable to gastroesophageal reflux disease.

Antibiotics are the cornerstone of treatment of neonates with sepsis [85]. Pentoxifylline is a nonsteroidal immunomodulating agent which has been used in several infectious, vascular, and inflammatory diseases in children [86]. Pentoxifylline is also used to treat neonatal sepsis [85]. A meta-analysis of 7 randomized controlled studies involving 439 newborns on the efficacy of pentoxifylline treatment for neonatal sepsis in China was carried out by Tian et al. [87]. Results of the meta-analysis demonstrated that pentoxifylline treatment had a positive impact reducing hospital stay and metabolic acidosis [85]. However, pentoxifylline provided no benefit on mortality for neonatal sepsis, disseminated intravascular coagulopathy and oliguria/anuria. The study by Tian et al. [87] had indeed several potential limitations that should be taken into account. The analysis included only 7 randomized controlled studies and 5 of them had small sample size (*n* < 100). Overestimation of the treatment effect is more likely in smaller trials compared with larger samples. Therefore, the heterogeneity of mortality in this meta-analysis was possibly caused by different schedules of pentoxifylline treatment.

## Neurology. 1- malignant spinal cord compression

Malignant spinal cord compression (MSCC) is one of the most feared complications of pediatric spinal cancer [88]. It is associated with poor prognosis as permanent paralysis, sensory loss, and sphincter dysfunction may develop. Very limited data are available on both incidence and etiology of MSCC in the pediatric population. To assess etiology, clinical presentation, and treatment of MSCC De Martino et al. evaluated retrospectively 44 pediatric patients with symptomatic MSCC who have been admitted to a children hospital since 2007 [89].

The median age at time of MSCC diagnosis was 52 months, with a peak in young patients (≤3 years). During the study period, the leading cause of MSCC in children aged less than 18 years were the extramedullary tumors (63.6%), in particular neuroblastoma (27.2%), followed by the Ewing sarcomas (15.9%), thus confirming previous data [90, 91]. Cord compression was the presenting feature of a new malignancy in 75% of patients. Motor deficit was the initial symptoms of spinal compression in all patients, while pain was present in about 60% of cases, followed by sphincteric deficit (43.2%), in according with De Bernardi’s data [92]. The median length of the interval between symptom onset and diagnosis widely ranged from 0 to 360 days in the entire population, however this interval was significantly shorter in patients with known neoplasia in comparison to patients with new diagnosis. Pre- and post-operative spine magnetic resonance imaging, i.e. the gold standard exam for the diagnosis, was performed in all cases, and most patients (95%) underwent neurosurgical treatment as first treatment. Treatment strategies differed widely among cancer types and study groups in the absence of optimal evidence-based treatment guidelines. It is well known that when diagnosis is uncertain, surgery provides an opportunity to biopsy the lesion in addition to treating the mass [93]. Patients with progression or relapsed disease showed a worst prognosis, while most patients (70.5%) were alive at 5 years after diagnosis. The natural history of MSCC in children is associated to permanent paralysis, sensory loss, and sphincter dysfunction, thus prompt diagnosis and correct management are needed to minimize morbidity [94].

## Nutrition. 1- undernutrition

Undernutrition in developing countries is a main problem of public health. Gizaw et al. [95] performed a systematic review and metanalysis including 16.473 children to assess the role of single and combined water, sanitation, and hygiene (WASH) intervention on nutritional status and growth. The results showed that WASH intervention significantly increased height-for-age z score and improve children nutrition in under-2 years children compared to under-five ones. Moreover, combined WASH interventions were more effective to improve child nutritional status compared to single interventions. This can be due to handwashing practice, drinkable water availability, and the subsequent reduction in infectious diseases transmission. A recent metanalysis conducted by Bekele et al. [96] confirmed that WASH interventions alone improved height-for-age z-score when delivered over 18–60 months and for children < 2 years. Integrated WASH with nutrition interventions may be effective in improving child growth outcomes. On the contrary, a trial conducted by Humphrey et al. [97] tested the independent and combined effects of improved WASH, and improved infant and young child feeding on stunting and anaemia in Zimbawe showing that elementary WASH interventions implemented in rural areas in developing countries are might not be enough to reduce stunting or anaemia and are might not be enough to reduce diarrhoea. Therefore, some authors [98] highlighted the importance of access to WASH as fundamental human right and although the study by Humphrey et al. did not show short-term effects on stunting, it cannot be underestimate a substantial intergenerational contribution to stunting. WASH in maternal education remains a strong predictor of child health outcomes, including stunting and nutrition.

## Respiratory diseases. 1- cystic fibrosis; 2- air pollution; 3- antibiotic prescriptions

Cystic fibrosis (CF) is the most common life-limiting autosomal recessive disease among people of European heritage that affects various organs of the body [99]. The most important cause of death in CF patients is due to lung involvement. Tabatabei et al. conducted a study to isolate and identify *Pandoraea species* bacteria from bronchoalveolar lavage and sputum samples of CF patients in Shiraz, Iran [100]. *Pandoraea species* are gram negative, motile, non-spore forming, rod shaped and oxidase positive, obligate aerobes bacteria, and have one polar flagellum [101]. Most of *Pandoraea species* are associated with lung infections in CF patients. In this study from 31 samples of bronchoalveolar lavage and sputum, 4 *Pandoraea* bacteria were isolated by culture method and identified by CRP using specific primer, then antibiotic susceptibility was also evaluated. Antibiotic treatment of infections caused by *Pandoraea species* is not as easy because resistance against a wide range of antibiotics such as gentamicin, amikacin and imipenem has been demonstrated and poor response to ciprofloxacin, trimethoprim-sulfumethoxazole, piperacillin and tetracycline is also frequently reported [102]. Conversely, another group reported that *P. sputorum* is susceptible to imipenem and resistant to meropenem [103]. This discrepancy has already been described by several authors and is due to the presence of meropenem-hydrolyzing β- lactamase [103]. According to the current study, ability to synthesize biofilm by *Pandorae*a isolates and resistance to antibiotics are very important issues. Biofilm formation indicates a protective mode of growth that allows microorganisms to survive in hostile environments [104]. In this context it seems essential to underline the importance of the combined use of the two CRP assays described for the identification of most *Pandoraea* species in bronchoalveolar lavage and sputum cultures of CF patients in order to reduce the misidentification of *Pandoraea* and the development of antibiotic resistance in CF [104].

The impact of air pollution on respiratory health has recently gained significant attention. Previous studies have found a correlation between air pollutants and multiple adverse effects on human health, in particular respiratory and cardiovascular health. In their study, Zhang et al. investigated the relationship between the air pollutant concentrations in Suzhou City, China, and respiratory tract infections in children of variable age caused by different pathogens using time series models with lagged effects [105]. They collected the clinical data from children hospitalized because of respiratory infections at a local Children’s Hospital during the years 2014 to 2016. They reported data from air quality measurement in Suzhou City in the same period and showed that PM_2.5_, PM_10_, NO_2_, SO_2_ and CO levels were significantly associated with respiratory tract infections in children under 3 years, with the largest effect sizes at a lag of 3 weeks. Other studies suggested that the length of this lag period may be greater for children under 5 years of age than for older children [106]. Notably, the multi-pollutant model found that PM_2.5_ level was significantly associated with viral respiratory in children under 7 months, and with bacterial respiratory infections in other age groups, while PM_10_ concentrations were associated with viral infections in preschool children. In a study conducted in Turin, Italy, Bono et al. showed an association also between O_3_ concentrations and higher numbers of emergency room visits by children presenting with respiratory problems [107]. The association between pollutant concentrations and viral and bacterial respiratory infections was stronger among children under 3 years than for older age groups, this being due to the faster respiratory rate of infants and young children, their immune system immaturity, and lower capacity to synthesize antibodies. All these factors may lead to greater susceptible to air pollutants. The PM_2.5_ and the O_3_ levels have been demonstrated to have the strongest influence on viral and *Mycoplasma pneumoniae* respiratory infections when multiple pollutants were tested together [105]. Taken together, these findings highlight the need for prompt interventions aimed to reduce air pollution, thus strengthening the importance of prevention, diagnosis, and treatment of respiratory infections in children.

Antimicrobials are the most widely prescribed drugs in children worldwide and almost half of antibiotic prescriptions in children are considered unnecessary [108]. Acute otitis media (AOM) and pharyngitis are two of the most common infections in pediatrics, and a main cause of antibiotic prescriptions [109]. Barbieri et al. conducted an observational, retrospective, outpatient study to describe the first-line treatment approaches to AOM and pharyngitis in primary care settings in Italy over 6 years [110]. Authors also included data about the prevalence of ‘wait and see’ for AOM and the differences in the prescriptions in cases with pharyngitis when the diagnostic tests are used. The data were collected using the Pedianet database that provides information from children aged 0–14 in Italy. Authors identified 120,338 children followed by 125 family pediatricians between January 2010 and December 2015 for a total of 923,780 person-years of follow-up. Of these, 30,394 children (mean age 44 months) received at least one diagnosis of AOM (*n* = 54,943) and 52,341 (mean age 5 years) had at least one diagnosis of pharyngitis (*n* = 126,098). A large proportion of children with AOM (82.5%) were treated with an antibiotic administered within 48 h. (mainly, amoxicillin and amoxicillin/clavulanate) and the “wait and see” approach was adopted only in 17.5% of cases. Despite guidance to use the ‘wait and see’ approach in the age group analyzed, this strategy is not often used for AOM, as previously noted in other studies conducted in hospital settings [111, 112]. Sixty-three percent cases of pharyngitis were treated, and among these, Group A β-haemolytic streptococcus pharyngitis, confirmed by rapid test (56%), were treated with amoxicillin. The ones not test-confirmed were treated with broad spectrum antibiotics [110].

The trend over time shows an increase in the broad spectrum antibiotic prescriptions in the last year (2015) [113]. The results of this study confirm once again that diagnostic uncertainty of AOM and pharyngitis is one of the determinants for antibiotics over prescription [114]. Finally, authors also found that perceived parental expectations of an antibiotic prescription and fear of under treatment is very commonly, according to other studies [115].

## Conclusions

In the second semester of year 2019, published papers have revealed new insights that are immediately helpful to improve care of children. They have enhanced our understanding of prevalence, natural course, mechanisms, symptoms, and treatment in many pediatric diseases. Moreover, reported information pave the way for further breakthroughs expected to improve diagnosis and clinical management of diseases in childhood.

## Data Availability

Data sharing is not applicable to this article as no datasets were generated or analyzed during the current study.

## Abbreviations

AOM: Acute otitis media
BPD: Bronchopulmonary dysplasia
CPAP: Continuous positive air pressure
CF: Cystic fibrosis
EDHS: Demographic and health survey
ELBW: Extremely low birth weight
FPIES: Food protein-induced enterocolitis Syndrome
GHD: Growth hormone deficiency
HCWs: Healthcare workers
hGH: Human growth hormone
HHHFNC: Humidified high-flow nasal cannula
INSURE: INtubation SURfactant Extubation
LBW: Low birth weight
MSCC: Malignant spinal cord compression
NCPAP: Nasal continuous positive airway pressure
NAVA: Neurally adjusted ventilatory assist
NIV: Non-invasive ventilation
NIPPV: Positive pressure ventilation
RDS: Respiratory distress syndrome
VR: Virtual reality
WASH: Water, sanitation, and hygiene
